# Discrepancies in the calibration of reaction rate analysers

**DOI:** 10.1155/S1463924680000284

**Published:** 1980

**Authors:** E. F. Legg, J. D. Cooper, M. R. Holland, J. Willis

**Affiliations:** 1 Department of Cinical Chemistry East Birmingham Hospital Birmingham UK; 2 Department of Biochemistry Coventry & Warwickshire Hospital Coventry UK; 3 Department of Chemical Pathology New Cross Hospital Wolverhampton UK; 4 Department of Medical Engineering East Birmingham Hospital Birmingham UK


### Discrepancies in the calibration of reaction rate analysers

E. F. Legg *
*Department of Clinical Chemistry, East Birmingham Hospital, Birmingham, England.*

J. D. Cooper
*Department of Biochemistry, Coventry & Warwickshire Hospital, Coventry, England.*

M. R. Holland
*Department of Chemical Pathology, New Cross Hospital, Wolverhampton, England.*

J. Willis
*Department of Medical Engineering, East Birmingham Hospital, Birmingham, England.*

### Introduction

Problems were encountered in ascertaining the cause of discrepancies between two LKB reaction rate analysers (RRA). One of the instruments consistently gave results which were 15% less than the values obtained from the other. The calibration procedure provided by the manufacturer, however, was not helpful in deciding at what level the fault was operating.

This paper describes the investigation of the factors responsible for the discrepancy. A calibration method is proposed which is designed to ensure better inter-laboratory agreement of enzyme analyses and which can be adapted to the calibration of any reaction rate analyser.

### Preliminary investigations

An initial investigation showed the calibration differences noted were not related to the reagent concentrations. Similarly, differences in pump volumes, incubator temperature, recorder voltages, chart speeds and cuvettes could not account for a difference between the instruments of greater than about 2%. The focussing of the incident light beam onto the circular cuvette was also found to be correctly aligned.

### Filters

A 5 nm difference was found between the peak absorbances of the original 340 nm filters used in the LKB reaction rate analysers. When both sets of filters were compared in the one instrument by means of an enzyme assay, they gave mean activities values 107.8 ± 5.6 (n = 10) and 103.8 ± 5.8 (n = 10) IU/L for the same test. This difference was significant at the 0.001 level and accounted for nearly 5% of the discrepancy between the instruments. All filters in subsequent studies were chosen with peak absorbances of 340 ± 2 nm.

### Absorbance scale errors

The LKB reaction rate analyser has two absorbance scales, one of 0.05 A and one of 0.2 A. Consistent differences were found between enzyme activities calculated using the 0.05 A scale and the 0.2 A scale on both instruments. Enzyme activities calculated using the 0.2 A scale were consistently lower (see Table 1). This scale error was significant at the 0.001 level and was confirmed on similar instruments in other laboratories.

### Temperature profile along the incubater tunnel

The mechanics of the LKB instrument are such that if the measuring time interval is set at 1 minute, the first cuvette reaching the measuring position has been in the thermostatted tunnel pre-incubating at 37°C for 7½ min. Subsequent specimens spend successively longer periods of time in the pre-incubation tunnel until a maximum of 15 min is reached. Thus several of the initial specimens spend a varying amount of time in the pre-incubation tunnel.

Evidence has been obtained to suggest that 7½ min in the pre-incubation tunnel is not sufficient time for the solution in the cuvette to equilibrate to 37°C, in that the enzyme activity is lower in the first few cuvettes compared with that in subsequent ones, and this is demonstrated by LKB1 in Figure 1. The company has modified the heating of the tunnel in their newer version of the instrument (LKB2). A booster heater is placed at the tunnel entrance which reaches a temperature of 50°C when a cold rack is inserted.

When replicate analyses were performed using this instrument (LKB2) it seemed that the cuvettes were now reaching the measuring position at too high a temperature since enzyme activity in the first few cuvettes was greater than the subsequent mean activity. This effect is demonstrated by LKB2 in Figure 1.

In addition, it is clear from Figure 1 that superimposed on this effect is another, whereby cuvettes placed at the front of the metal racks in LKB2 tend to give rise to higher enzyme activities than those at the rear of the rack.

### Development of calibration procedure

The combined effect of the variations between the two analysers noted above was sufficient to account for approximately one third of the 15% discrepancy originally noted.

At this stage it was considered appropriate to calibrate each instrument against an absolute standard.

### Naphthol green procedure

The LKB company has published a method utilising naphthol green [1] for the calibration of the reaction rate analyser. Briefly, a dilution of a stock solution of naphthol green is prepared and filtered to give a series of solutions varying in absorbance from 0.4 to 0.6, so that their absorbance

**Table 1. Demonstrating the mean and standard deviation of replicate LD activities (IUL) obtained using the 0.05 and 0.20 absorbance scales from two LKB reaction rate analysers.**

| | 0.05 Scale Mean ± 1 SD n = 20 | 0.20 Scale Mean ± 1 SD n = 20 | Probability |
|---|---|---|---|
| LKB 1 | 731 ± 24 | 671 ± 15 | <0.001 |
| LKB 2 | 636 ± 16 | 586 ± 13 | <0.001 |

*To whom requests for reprints should be sent.

differences span the higher absorbance scale of 0.2A. The absorbance differences obtained at 340 nm are compared with those obtained using a high resolution spectrophotometer and should agree within ± 5% according to the manufacturers specification [2].

The term high resolution spectrophotometer is used here to define a spectrophotometer possessing a band width of 1 nm or less [2]. A Pye Unicam SP1800 (Cambridge, England) fulfills this criterion and was used in this study. Each measurement was performed in triplicate. Good agreement was not obtained, the values differing by 10–30%.

### The use of NADH as chromophore rather than naphthol green

Naphthol green is not the most suitable chromophore to use for calibration purposes since most enzymic reactions are linked to NADH. It must be remembered that all kinetic enzyme analyses linked to the formation or disappearance of NADH rely on the accuracy of the formula:-

$$\text{Enzyme activity (IU/L)} = \frac{A/\text{min}}{6220} \times \frac{TV}{SV} \times 10^6$$

where A/min = change in absorbance
TV = Total volume
SV = Sample volume
6220 = Accredited molar absorbance NADH [3]
$10^6$ = Conversion factor from mol to $\mu$mol

Any error, therefore, in the apparent molar absorbance of NADH will give rise to errors in the enzyme activity obtained.

The apparent absorption of a chromophore in a filter photometer depends on the ratio of the half bandwidth of the filter to the half bandwidth of the chromophore, i.e. the relative band width (RBW).

Table 2 which is modified from that of Burke *et al* [4] illustrates the influence of relative band width on the apparent absorbance of a chromophore and shows that the greater the relative band width, the greater the discrepancy between true and observed absorbance.

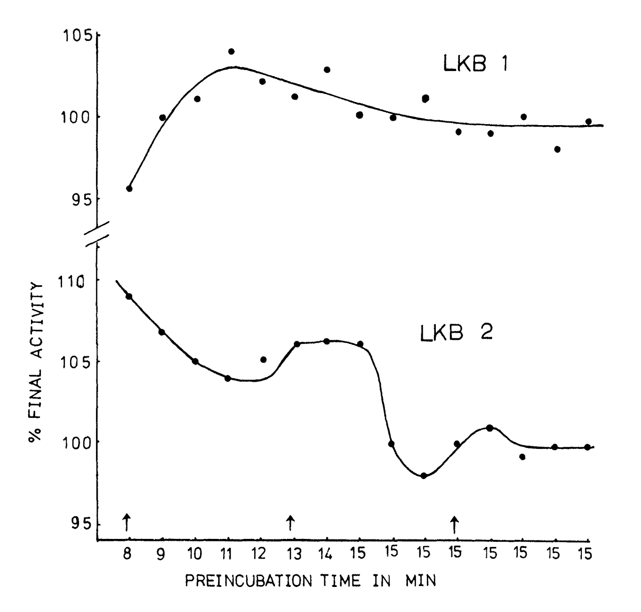

*Figure 1. Demonstrating effect of time of preincubation on enzymic activity using LKB1 and LKB2 for a series of replicate analyses. Each point is the mean of two successive readings. The arrows ↑ indicate the reading of the first cuvette in the metal rack.*

The absorbance spectra of a 340 nm LKB interference filter, naphthol green and NADH solutions are illustrated in Figure 2.

Since the half bandwidth of NADH absorption is about 50 nm and the half bandwidth of the filter is 10 nm, the relative band width for NADH and the filter is 0.20, producing an apparent absorbance 98.19% of the true absorbance (Table 2). The use of naphthol green however, because of its extremely flat absorbance curve, would give rise to an apparent absorbance of 100% of the true absorbance and thus not reflect the apparent molar absorptivity of NADH under the conditions of the test. Hence NADH and not naphthol green should be used as the chromophore of choice when calibrating the LKB or any other type of enzyme reaction rate analyser.

### Proposed calibration procedure using NADH

Dilutions of a stock solution of NADH (24 milli molar in tris buffer pH 7.4) were prepared to give approximate absorbances of 0.003, 0.05, 0.10, 0.15 and 0.20. The absorbances of three separate portions of each solution were measured carefully in a spectrophotometer at 37°C.

The absorbances were again measured in triplicate, using the LKB reaction rate analyser in the 'hold background'

**Table 2. Dependence of % true absorbance on relative band width under idealised conditions [4]**

| Relative bandwidth | % true absorbance |
|---|---|
| 0.03 | 99.95 |
| 0.07 | 99.77 |
| 0.10 | 99.54 |
| 0.20 | 98.19 |
| 0.30 | 96.04 |
| 0.40 | 93.21 |
| 0.50 | 89.87 |

$$\%\ \text{true absorbance} = \frac{\text{observed absorbance}}{\text{True absorbance}} \times 100$$

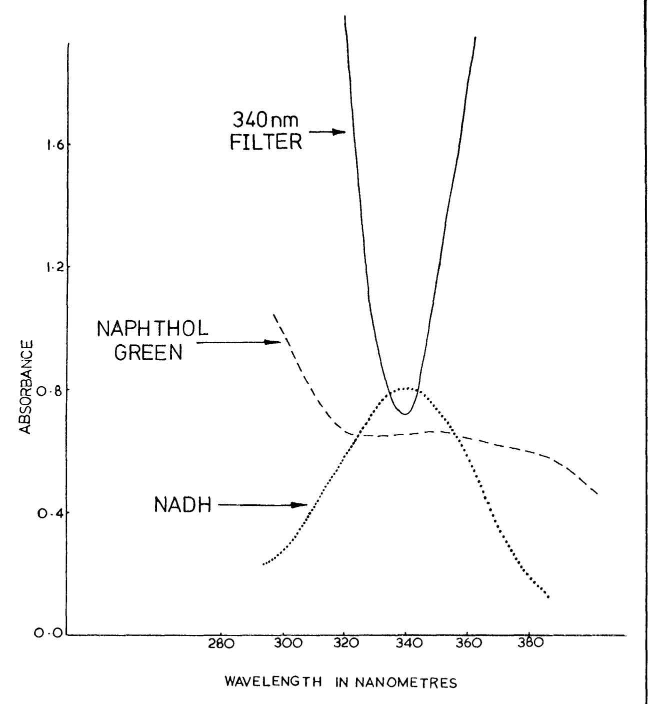

*Figure 2. Absorbance spectra of NADH, naphthol green and an LKB interference filter of 340 nm.*

mode at 37°C [1]. A typical example of the recorder trace obtained is shown in Figure 3.

Because of the indeterminate nature of the zero [1] on the LKB instrument, it is mean absorbance *differences* of the solutions, rather than the absolute absorbances, which are calculated and compared with those obtained from the high resolution spectrophotometer. An X–Y plot of the absorbance differences is then drawn. Assuming the results from the high resolution spectrophotometer are true absorbances, a value for the apparent molar absorption coefficient of NADH can be obtained (Figure 4) by multiplying the slope by 6220 [3]. The high resolution spectrophotometer was assumed to give true absorbance readings, since the wavelength accuracy as adjudged by a Holmium filter was better than 1 nm and the absorbance of a 1 g/l solution of potassium dichromate was 10.80. (The National Bureau of Standards (Washington, DC) ascribe an absorbance of 10.71 [4] to this solution).

Thus LKB1 gives rise to an apparent absorbance coefficient for NADH of 0.91 x 6220 = 5660, and LKB2 gives rise to an apparent absorbance coefficient for NADH of 0.80 x 6220 = 4976, i.e. a difference of 11%. This difference, coupled with the 5% discrepancy noted previously as a result of the problem with the interference filters accounts for the initial 15% discrepancy observed.

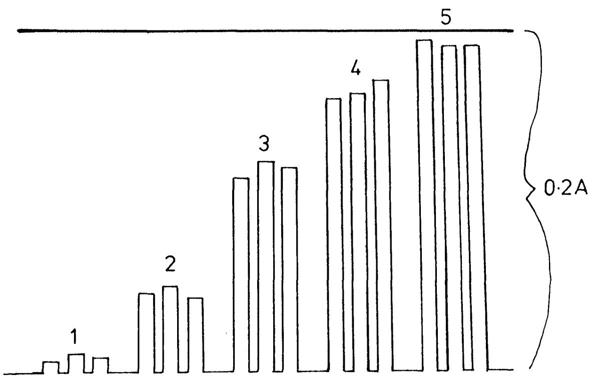

*Figure 3. A typical LKB recorder trace obtained using the proposed calibration procedure. The peaks represent the absorbances due to a series of 5 solutions of NADH measured in triplicate. The mean height of each triplet is measured. The mean absorbance differences between solution 5 and solutions 1, 2, 3 and 4 are then calculated and compared with the absorbance differences obtained using a high resolution spectrophotometer.*

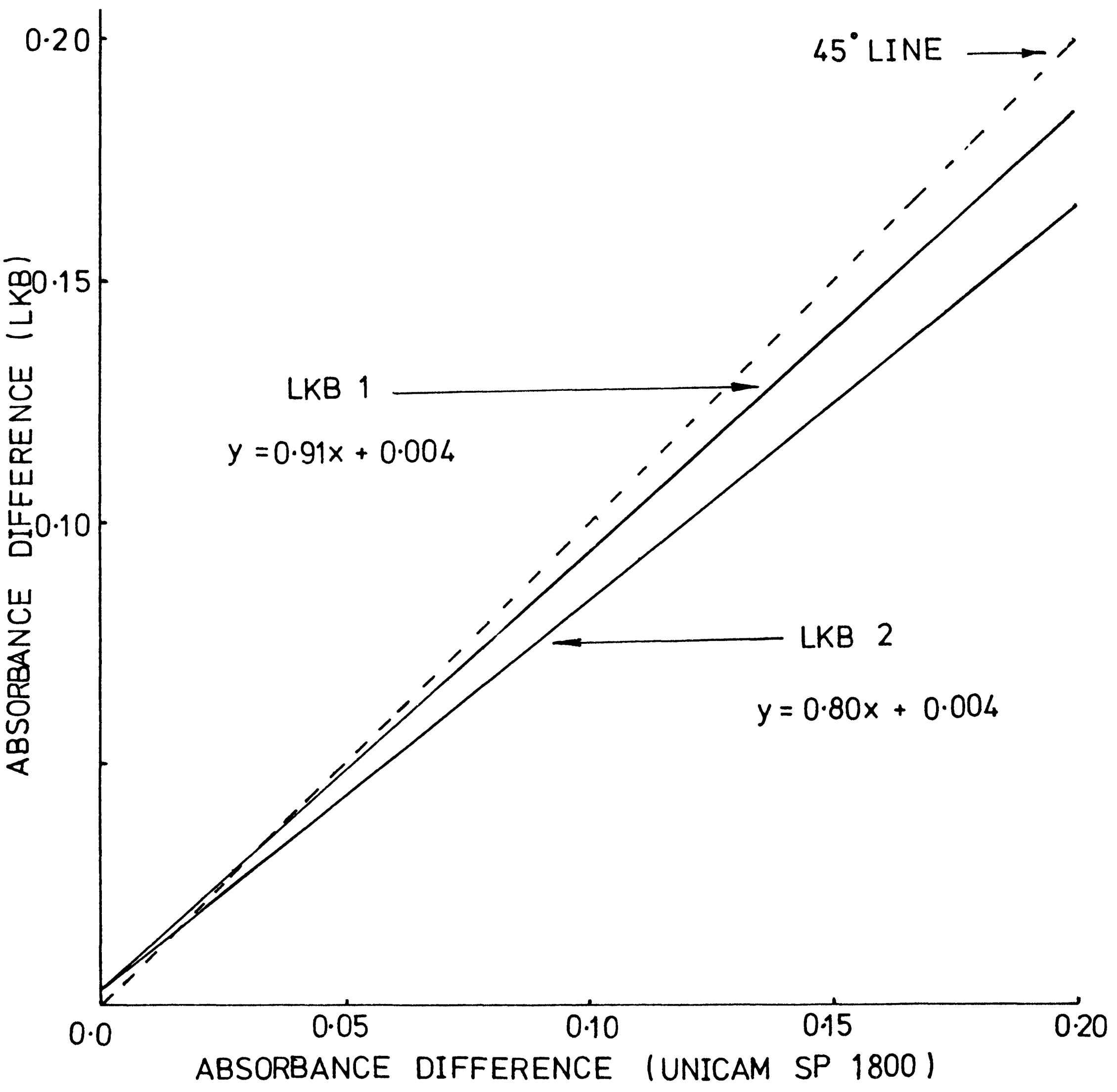

*Figure 4. X–Y Plot of the absorbance differences obtained from LKB 1, LKB 2 and a Pye Unicam SP1800 using the proposed calibration procedure. The same filters were used in both instruments.*

It was considered at this stage that the problem that remained was electronic rather than one associated with chemistry, optics or mechanics. The manufacturers were contacted and agreed to provide details of an electronic calibration procedure used by their engineers in the field.

### Electronic calibration

This procedure involves adjustments designed to set voltages in the feedback loop around the detector unit. These adjustments are directly responsible for the subtraction of the initial background peak from the reaction curve and the initiation of absorbance monitoring from a reproducible zero baseline. Settings include adjustment of reference and absolute ground voltages in the feedback loop operating in both increasing and decreasing modes.

Further adjustments alter the span and linearity relationship between the output signal from the instrument and the recorder registration level, and also the switching point and ratio balance between the ranges of 0.05 and 0.20 absorbance units. Some of these settings require a 'compromise' between two required voltage levels and for reasonable accuracy a volt-meter with a minimum of four digit resolution should be used.

This procedure was carried out on both LKB reaction rate analysers and absorbance difference was plotted against spectrophotometer absorbance difference as described previously. The slopes for both LKB1 and LKB2 were now almost identical (Figure 5). This indicates that the instruments had been calibrated to give the same result. This was confirmed by analysis of 10 replicate serum samples for lactate dehydrogenase activity of mean values of 676 ± 11 IU/L and 676 ± 19 IU/L were obtained respectively for LKB 1 and 2.

### Discussion

Discrepancies between instruments, such as those in question, can arise either from chemical or instrumentation faults. In this case the recommended method of instrument calibration was unable to differentiate the faults and so a number of possible causes were investigated. The differences found between filters are well recognised and this variable was eliminated in further studies; however the disparity between the absorbance scales and the influence of the heating mechanism of the incubator tunnel on the reaction are not well known and need to be recognised and corrected.

Although the LKB instrument incorporates a basic filter photometer, it should operate within the manufacturer's specification of ± 5%. However, using the naphthol green calibration procedure recommended by the company, this specification was rarely achieved and a different calibration procedure was adopted.

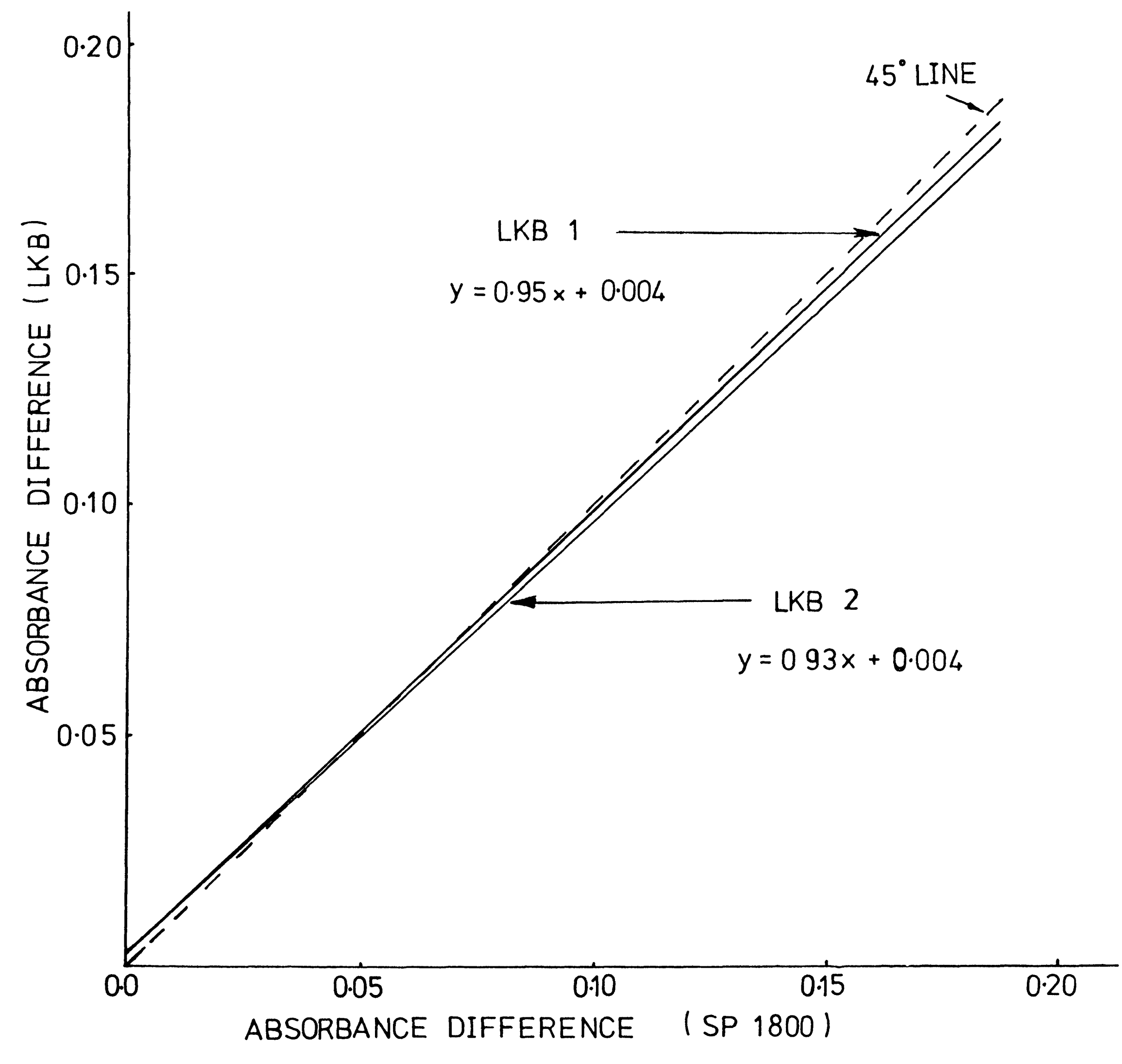

*Figure 5. Demonstrating agreement of apparent molar absorptivities for NADH on LKB 1 and LKB 2 after the instruments were electronically calibrated. The same filters were used in both instruments.*

Many enzyme assays are linked to the formation or utilisation of NADH so that reaction rates can be monitored at 340 nm. The absorbance spectrum of naphthol green is sufficiently different from that of NADH to render calibration using this substance undesirable, and NADH or the particular chromophore used to monitor the reaction, e.g. p-nitrophenol, should be used.

The new calibration procedure confirmed the extent of the disparity between the two instruments noted when performing enzyme analyses and supported the view that the problem was one of instrumentation, possibly electronic in origin. An electronic calibration procedure produced by the company was successful in bringing the instruments back into specification and cured the problem of the disparity between the 0.05 and 0.20 absorbance scales.

Exactly what is accomplished when electronic calibration is carried out remains uncertain. Certainly such items as the ratio of the two absorbance scales and linearity across the recorder are correctly adjusted during this procedure, but the manner in which the photocell output is linked to true absorbance is not clear. In the light of the authors' experience, it is difficult to avoid the conclusion that electronic calibration merely succeeded in ensuring that both instruments investigated produced the same result. It may not ensure that the output from an instrument is linked to true absorbance.

For this reason, a correction factor may still need to be applied to the results obtained after electronic and absorptiometric calibration has been carried out. It is suggested that results which are within ± 5% of the true absorbance need not be corrected, while instruments operating outside of these limits should have a correction factor applied until they can be serviced and the error corrected. This may well depend on the type of analysis being performed on the instrument.

The calibration procedure outlined could be performed at intervals of about six months. It can be adapted to calibrate any other type of reaction rate analyser and has been used successfully in calibrating both the AKES (MSE Scientific Instruments, Manor Royal, Crawley, West Sussex), and Centrifichem (Union Carbide (U.K.) Ltd., Meteor House, White Lion Road, Amersham, Bucks) systems. If such a proposal were generally adopted, better inter-laboratory agreement of enzyme results should occur. For future instruments, recommendations have been made which set very high specifications [5] and which should render future calibration a more precise and rapid exercise.

ACKNOWLEDGMENT
The authors would like to thank Dr. H. G. Sammons for his interest and advice.

REFERENCES

[1] Instruction manual to Reaction Rate Analyser 2086 Appendix VIII LKB Produkter A.B. Sweden.
[2] Tietz, N. W. (Ed.), 1976, Fundamentals of Clinical Chemistry, 2nd Edition, W. B. Saunders & Co. Philadelphia.
[3] Moss, D. W., 1976, News Sheet No. 159, Association of Clinical Biochemists.
[4] Burke, R. W., Deardorff, E. R. and Menis, O. 1972, Liquid Absorbance Standards, *J. Research. Nat. Bureau of Standards A. Physics and Chemistry*, 76A, 469.
[5] Instrumentation Guidelines Study Group, 1977, Guidelines for photometric instruments for measuring enzyme reaction rates, *Clinical Chemistry*, 23, 2160-2162.

### An evaluation of the Nova 2 ionised calcium instrument

**J.A. Fyffe, A.S. Jenkins and H.N. Cohen**
*University Department of Medicine, Royal Infirmary, Glasgow G4 OSF*

**F.J. Dryburgh and M.D. Gardner**
*Department of Biochemistry, Royal Infirmary, Glasgow G4 OSF*

### Introduction

The hypothesis of McLean and Hastings [1] that free or ionised calcium is the physiologically active fraction of plasma calcium is now well accepted. Various methods of measuring this have been used including bioassay [1,2] bioluminescence [3,4] ultra-filtration [5] and ion-selective electrodes [6-13] Ion-selective electrodes have been available for nearly twenty years and specific versions for use in the clinical laboratory for more than ten years. Many of these however have serious shortcomings. They are difficult to set up and have a short membrane life. Once set up they provide a simple and rapid measurement of ionised calcium.

The Nova 2 instrument shown in Figure 1 is manufactured by Novabiomedical, Newton, Mass. U.S.A. and marketed in the United Kingdom by American Hospital Supply (U.K.) Ltd., Didcot, Oxon. The electrode assembly consists of a calcium selective electrode and a silver-silver chloride reference electrode with a KCl bridge. The calcium selective electrode is housed in a plastic box containing internal filling solution (calcium chloride) in a gel form. The standard and test solutions flow through the teflon tube which passes through the gel.

In the teflon tube is an ion-selective window which acts as a membrane. The inner surface of the window is coated with a calcium polyphosphate ion exchanger in a non-aqueous medium. An internal silver/silver chloride electrode connects the ion-selective electrode by a silver wire to the electronic circuit.

The silver/silver chloride reference electrode consists of a silver wire embedded in a silver chloride pellet. The internal reference solution (2 M KCl) flows past this to meet the sample stream in a dynamic liquid junction. The reference electrode also incorporates a pair of platinum electrodes which sense the presence of air or liquid and are used by the computer to monitor cycle performance. The electrodes are mounted by a simple plug-in device in a heated aluminium block which is maintained at a temperature of 37°C. A calcium electrode, a reference electrode and a spare calcium electrode (these are guaranteed for six months use provided Nova fluids packs are used) are supplied with the instrument.

The functions of the analyser are controlled by a small in-built computer and selection of the 'calibrate' or 'analyse' cycle is by simple push buttons. The instrument has two operating modes, 'stat' and 'stand by'. In the 'stat' mode the

